# Investigating the interplay between social exclusion and meaning in life: the catalytic influence of psychological flexibility and basic psychological needs

**DOI:** 10.1186/s40359-026-04616-0

**Published:** 2026-04-28

**Authors:** Qing Li, Zongzheng Zhang, Meiqi Ge, Yi Wang, Xiaochen Wang

**Affiliations:** 1 School of Publishing, Communication University of Zhejiang, Hangzhou, 310018 China; 2 AI Lab for International Communication, Communication University of Zhejiang, Hangzhou, 310018 China; 3 School of Business Administration, Zhejiang Gongshang University, Hangzhou, 310018 China; 4 Faculty of Arts, The University of Hong Kong, Hong Kong, 999077 China; 5 Zhejiang Tianjun Asset Management Co., Ltd, Hangzhou, 310000 China; 6 Inamori Business School, Zhejiang Gongshang University, Hangzhou, 310018 China

**Keywords:** College students, Social exclusion, Meaning in life, Basic psychological needs, Psychological flexibility

## Abstract

**Background:**

Although prior studies have demonstrated that social exclusion adversely influences individuals’ sense of meaning in life, the underlying mediating mechanisms and moderating boundaries have not been fully examined. Accordingly, the present study investigated whether basic psychological needs mediate the relationship between social exclusion and meaning in life, and whether this mediating pathway is moderated by psychological flexibility.

**Methods:**

A three-wave, time-lagged quantitative design was employed with a sample of 1,056 Chinese college students. Participants completed validated questionnaires assessing social exclusion, meaning in life, basic psychological needs, and psychological flexibility.

**Results:**

Social exclusion was negatively associated with meaning in life, and basic psychological needs served as a significant mediator in this relationship (indirect effect = − 0.21). Psychological flexibility significantly moderated the association between social exclusion and basic psychological needs (interaction effect = 0.11, *p* < 0.01), and also moderated the mediating effect of basic psychological needs on meaning in life.

**Conclusion:**

Findings indicate that social exclusion may erode college students’ sense of life purpose by undermining the satisfaction of basic psychological needs. This underscores the importance of providing adequate social resources and opportunities to meet diverse student needs and foster interpersonal support. Moreover, targeted psychological training should be prioritized for students exhibiting low psychological flexibility. Implementing evidence-based interventions to enhance adaptability and positive cognitive reappraisal may help students accept challenging circumstances, shift toward constructive thinking patterns, and strengthen their sense of meaning in life.

## Introduction

The quest for meaning in life is a fundamental psychological endeavor that serves as a compass for goals and a primary source of self-worth [1]. However, amidst intensifying competitive pressures, many Chinese college students exhibit a “meaning deficit,” which manifests as existential emptiness and academic burnout [2].While meaning in life functions as a critical protective buffer against such psychological distress [3, 4], research addressing its antecedents and formation mechanisms remains limited. Therefore, it is an imperative scholarly endeavor to systematically investigate these predictors and explore effective intervention.

As inherently social beings, humans possess a fundamental drive for connection and belonging, which constitutes the psychological bedrock for the pursuit of meaning [5]. Among college students, social exclusion represents a pervasive and detrimental interpersonal stressor. It systematically thwarts basic psychological needs—specifically, the needs for autonomy, competence, and relatedness—thereby eroding the sense of meaning in life [6]. However, the magnitude of this effect is not uniform; it is significantly contingent upon an individual’s psychological flexibility. Psychological flexibility is defined as the capacity to persist in or change behavior in the service of chosen values, even in the presence of difficult experiences [7]. It is hypothesized that individuals with lower psychological flexibility experience more acute need frustration in response to exclusion, precipitating a sharper decline in meaning. Conversely, those with high psychological flexibility can mitigate the degree of need frustration through adaptive cognitive and behavioral responses, thereby buffering the deleterious impact of social exclusion on meaning [7].

Grounded in this theoretical framework, the present study aims to systematically elucidate the mechanism linking social exclusion to college students’ sense of meaning in life. Specifically, this research focuses on the mediating role of basic psychological needs and the moderating effect of psychological flexibility within this mediated pathway.

### Social exclusion and meaning in life

Existential psychology posits that individuals seek “meaning” through interpersonal interactions [8] and endeavor to establish and maintain meaningful social connections across the lifespan. Robust social support serves as a critical contextual factor, fostering the development and enrichment of meaning in life. King [9] identified three fundamental pathways to cultivate meaning: *belonging* (close and supportive relationships), *action* (engagement in purposeful activities), and *understanding* (developing insight into oneself and the world). Belonging, as a form of social support, plays a pivotal role in sustaining and constructing meaning in life.

In contrast, social exclusion—conceptualized as the rejection of an individual by others and the denial of participation in group activities—undermines and obstructs the fulfillment of belonging and affiliation needs [10]. Prior research indicates that socially excluded individuals often experience a lack of social connectedness, which may increase the likelihood of deviant behaviors [11]. Williams’ temporal need–threat model provides a theoretical framework for understanding individual responses to exclusion [12]. The model asserts that social exclusion diminishes core psychological resources, including perceived control, self-esteem, belonging, and the sense of meaning, thereby heightening vulnerability to deviant conduct. Prolonged exclusion has been shown to exhaust coping capacities and weaken one’s ability to navigate social environments to restore belonging, ultimately precipitating intense feelings of depression, helplessness, and existential meaninglessness [13].

Against the backdrop of rapid technological and societal advancement, college students navigate a critical developmental stage characterized by heightened psychological and physiological plasticity. This demographic encounters multifaceted stressors stemming from academic demands, interpersonal dynamics, and uncertainties regarding future career prospects [14]. Compared to children and older adults, late adolescents and emerging adults exhibit heightened sensitivity to interpersonal neglect, rendering them particularly susceptible to the deleterious effects of social exclusion. Moreover, social exclusion constitutes a potent determinant of mental health outcomes. Empirical evidence indicates that even incidental instances of exclusion can precipitate acute negative affective states, including anger, sadness, anxiety, and depression [11]. Furthermore, social exclusion has been shown to compromise physical health by inducing physiological stress responses, such as elevated serum cortisol concentrations [15]. Research further reveals that ostracized individuals often employ social withdrawal as a defensive strategy to mitigate distress; however, this withdrawal is frequently accompanied by profound alienation, helplessness, and a pervasive sense of meaninglessness [13]. Drawing upon Baumeister’s theoretical framework [16], which posits that social exclusion fundamentally undermines the pillars of meaning in life, we propose the following hypothesis:

- Hypothesis 1: Social exclusion is negatively related to meaning in life.

### The mediating role of basic psychological needs

Deci and Ryan’s Basic Psychological Needs Theory posits the existence of three fundamental human needs: competence, autonomy, and relatedness [17]. These needs function as essential psychological nutrients; when satisfied by the external environment, they facilitate optimal functioning, positive development, and psychological growth. Conversely, if these needs are thwarted, individual development is significantly hindered. Among these, the need for relatedness—characterized by the desire to be acknowledged, accepted, and to establish stable, positive bonds with others—is paramount. Empirical research indicates that peer interactions play a pivotal role in the satisfaction of these needs. Specifically, when college students experience peer exclusion, it engenders negative interpersonal experiences that frustrate overall basic psychological need satisfaction [18] and specifically impede the sense of belonging [19]. Furthermore, literature suggests that interpersonal relationships are a critical environmental determinant of one’s sense of meaning in life. Consequently, social exclusion may severely compromise the need for belonging, thereby precipitating a diminished sense of meaning in life.

Basic Psychological Needs Theory further posits that individuals flourish when their external environment supports the satisfaction of these needs; conversely, when these needs are thwarted, development is significantly compromised. The dynamic processes of self-improvement and psychological integration are intrinsic to the construction of meaning in life [20, 21]. When the social environment facilitates need satisfaction, individuals are more likely to perceive their lives as meaningful and actively seek experiences that reinforce this sense of purpose [22]. In contrast, individuals experiencing low levels of need satisfaction often exhibit maladaptive outcomes, including depression, apathy, and even suicidal ideation and behaviors [23].

In light of these findings, the present study hypothesizes that basic psychological needs serve as a fundamental internal mechanism mediating the relationship between social exclusion and meaning in life. Specifically, it is posited that social exclusion thwarts the satisfaction of college students’ basic psychological needs, which in turn diminishes their sense of meaning in life. Accordingly, the following hypothesis is proposed:

- Hypothesis 2: Basic psychological needs mediate the relationship between social exclusion and meaning in life.

###  The moderating role of psychological flexibility

Psychological flexibility comprises a repertoire of cognitive and behavioral skills that underpin holistic well-being, resilience, and efficacy [24]. Individuals exhibiting high psychological flexibility possess the capacity to accept and attend to present-moment experiences, adopt a self-as-context perspective, and respond adaptively to adverse emotional events. Conversely, individuals characterized by low psychological flexibility often exhibit cognitive rigidity, heightened susceptibility to negative affect, and difficulty recovering from setbacks. Functioning as a pivotal situational moderator of risk [25, 26], psychological flexibility facilitates resilience in the face of adversity [27]. Empirical evidence further indicates that psychological flexibility is a primary determinant of psychopathology and psychological distress [28]. As the cornerstone of Acceptance and Commitment Therapy (ACT), this construct enables individuals to adapt to fluctuating situational demands, restructure maladaptive thought patterns, and deploy effective coping strategies [29]. Within the context of social exclusion—a potent situational risk factor—college students with high psychological flexibility are better equipped to navigate stress. Their heightened resilience acts as a buffer, mitigating the detrimental impact of exclusion on the satisfaction of basic psychological needs. In contrast, students with low psychological flexibility may become entrapped in rigid patterns of negative emotion and cognition, making it difficult to extricate themselves from the adverse effects of exclusion. Accordingly, the following hypothesis is proposed:

- Hypothesis 3: Psychological flexibility moderates the relationship between social exclusion and basic psychological needs. Specifically, the negative association between social exclusion and basic psychological needs will be weaker for individuals with high psychological flexibility compared to those with low psychological flexibility.

Integrating Hypothesis 2 and Hypothesis 3 suggests a moderated mediation model. It is posited that the indirect effect of social exclusion on meaning in life, transmitted through basic psychological needs, is contingent upon the individual’s level of psychological flexibility. Specifically, for students with high psychological flexibility, the detrimental impact of social exclusion on basic psychological needs is attenuated; this preservation of need satisfaction, in turn, sustains a higher sense of meaning in life. Consequently, the indirect negative effect of social exclusion on meaning in life via basic psychological needs is expected to be less pronounced when psychological flexibility is high. In summary, the following moderated mediation hypothesis is proposed:

- Hypothesis 4: Psychological flexibility moderates the indirect effect of social exclusion on meaning in life via basic psychological needs, such that this indirect effect is weaker when psychological flexibility is high.

**Fig. 1:**
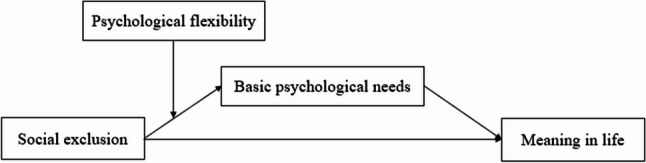
Conceptual model

The overall theoretical framework of the study is presented in Fig. 1.

## Method

### Sample and procedure

The study employed a cluster sampling method with online questionnaires to investigate the relationship between social exclusion, psychological flexibility, basic psychological needs, and meaning in life. Participants were recruited through public courses at two universities in Zhejiang province, China. Completing the surveys was voluntary to assure anonymity. After checking that participants understood the instructions, they completed the survey independently.

Due to common method bias that might exaggerate the correlation between variables and lower the precision of results, we gathered data in three phases. For the first time, participants were asked to complete the questionnaires on social exclusion, psychological flexibility, and demographics. We gathered a sample of 1313 individuals. Three weeks later, the same procedures were followed to distribute questionnaires for the second time, in which respondents were required to rate their degree of basic psychological needs. At this stage, 1199 valid questionnaires were gathered. Approximately Three weeks later, for the third time, respondents were asked to complete a measure of meaning in life. After eliminating invalid data and matching the three data waves, we eventually maintained 1056 respondents in the sample. Overall, 80.43% of requested data were returned.

Among the valid questionnaires, 469 males (44.41%) and 587 females (55.59%) participated, with 316 freshmen (29.92%), 326 sophomores (30.87%), 218 juniors (20.64%), and 196 seniors (18.56%).

### Variables measurement

#### Social exclusion

The study utilized the Chinese version of the Ostracism Experience Scale for Adolescent Scale (OES-A) to assess the extent of social exclusion experienced by students. The OES-A was originally developed by Gilman [30] and subsequently revised by Zhang et al. [31]. The scale contained 11 items, measuring two dimensions of neglection and rejection. Respondents rated their agreement level on a 5-point Likert scale, ranging from 1 (strongly disagree) to 5 (strongly agree). A sample item is “In general, others treat me as if I am invisible.” (Cronbach’s *α* = 0.81).

#### Basic psychological needs

The Basic Psychological Needs Scales (BPNS) for students were used to examine the basic psychological needs in the study. The BPNS was initially complied with by Gagne [32] and later revised by Liu et al. [33]. The scale comprised 21 items that were divided into three dimensions: autonomy needs, relationship needs, and competence needs, with 7, 6, and 8 items, respectively. Respondents indicated their level of agreement on a 5-point Likert scale ranging from 1 (strongly disagree) to 5 (strongly agree). Sample items included, “I feel I can decide how to live my life” (Cronbach’s *α* = 0.81).

#### Meaning in life

The study adopted the Meaning in Life Questionnaire (MLQ) by Steger et al. [34] to assess the perception of meaning in life and the drive to pursue meaning in life among participants. The questionnaire comprised two subscales, namely, the presence of meaning and the pursuit of meaning, each consisting of 5 items. Examples included “I understand my life’s meaning” and “I am looking for something that makes my life feel meaningful”. Participants were required to rate their level of agreement on a five-point Likert scale, ranging from 1 (strongly disagree) to 5 (strongly agree), with higher scores indicating a greater sense of meaning in life (Cronbach’s *α* = 0.80).

#### Psychological flexibility

The present study evaluated psychological flexibility using the Acceptance and Action Questionnaire-2nd Edition (AAQ-II), which was originally developed by Bond et al. [35] and adapted by Cao et al. [36] with comprehensive reliability assessment in China. The scale comprised 7 items, with a five-point response format ranging from 1 (strongly disagree) to 5 (strongly agree). Reverse scoring was applied to higher scores, indicating greater psychological flexibility. A sample item from the AAQ-II is “It seems like most people are handling their lives better than I am” (Cronbach’s *α* = 0.80).

#### Control variables

In the current study, we controlled demographic factors, including gender and grade. Gender was dichotomized as 1 for male and 2 for female, while grade level was classified into four categories: 1 for freshman, 2 for sophomore, 3 for junior, and 4 for senior.

### Analytic strategy

Our research employed SPSS 24.0 and Mplus for data analysis and implemented Bootstrapping through the PROCESS V3.3 macro program of SPSS. Initially, a confirmatory factor analysis was performed to evaluate the validity and determine the Cronbach’s *α* to assess internal consistency. Secondly, descriptive statistics and Pearson correlations were computed to examine the relationships among the research variables. Thirdly, model 4 in the SPSS PROCESS macro was used to evaluate the mediating effect of basic psychological needs. In contrast, Model 7 was employed to investigate the moderating effect of psychological flexibility on the indirect relationship between social exclusion and meaning in life. Furthermore, demographic characteristics were controlled while assessing the mediating and moderating effects. Bootstrap confidence intervals (CIs) evaluated the significance of the effects of Model 4 and Model 7 based on 5,000 random samples. An effect was deemed significant if the CIs did not include 0.

## Results

### Confirmatory factor analysis

Using the Harman one-factor test, we first examined the common method bias [37]. The results showed that the mutation rate explained by the first factor was 16.50%. This value was less than the critical value of 40%, suggesting that this study did not deviate significantly from the common method.

Further, we compared the baseline four-factor model with the three-factor, two-factor, and one-factor models. The results are shown in Table 1. The comparison was based on the changes in chi-square (i.e., *χ*^*2*^), comparative fit index (CFI), tucker-lewis index (TLI), and root mean square error of approximation. The comparison revealed that the four-factor model (with *χ*^*2*^*/df* = 1.89, TLI = 0.90, CFI = 0.90, and RMSEA = 0.03) fit the data better than other alternative models [38, 39], supporting the discriminatory validity of the constructs. This indicated the capacity of the respondents involved in the investigation to distinguish between the focal constructs.

**Table 1:** Results of confirmatory factor analysis

Measurement Models	χ^2^	df	χ^2^/df	RMSEA	TLI	CFI
Model 1: One-factor (combined all items into one factor)	5419.41	1080	5.02	0.06	0.54	0.56
Model 2: Two-factor (combined SE and BPN into one factor, and combined ML and PF into one factor)	5173.18	1079	4.79	0.06	0.57	0.58
Model 3: Three-factor (combined SE and BPN into one factor)	2839.80	1077	2.64	0.04	0.81	0.82
Model 4: Four-factor	2030.82	1074	1.89	0.03	0.90	0.90

*SE *Social exclusion, *BPN *Basic psychological needs, *ML *Meaning in life, *PF* Psychological flexibility

### Descriptive statistics and correlation analysis

Table 2 displays the variables’ means, standard deviations, Cronbach’s *α*, and correlation coefficient. Social exclusion was significantly negatively associated with basic psychological needs (*r* = − 0.49, *p* < 0.01) and with meaning in life (*r* = − 0.34, *p* < 0.01). In contrast, basic psychological needs were positively correlated with meaning in life (*r* = 0.48, *p* < 0.01). Hence, these findings tentatively validated the forthcoming regression analysis.

**Table 2:** Descriptive statistics of and correlations among study variables

Variable	M	SD	1	2	3	4	5	6
1 Gender	1.56	0.50						
2 Grade	2.28	1.08	0.04					
3 Social exclusion	2.56	0.47	0.005	− 0.01	(0.81)			
4 Basic psychological needs	3.41	0.38	0.01	0.04	− 0.49^**^	(0.81)		
5 Psychological flexibility	2.99	0.57	0.04	0.04	0.10^**^	0.07^*^	(0.80)	
6 Meaning in life	3.55	0.50	− 0.01	0.01	− 0.34^**^	0.48^**^	0.03	(0.80)

*p < 0.05; **p < 0.01

### Hypotheses testing

To assess the mediating effect of basic psychological needs on the relationship between social exclusion and meaning in life, we applied model 4 from Hayes’ SPSS Macro PROCESS [40]. Table 3 presents the results of the mediation analysis and the relationships among the variables.Results showed that social exclusion negatively predicted meaning in life (*β* = − 0.14, SE= 0.03, *p* < 0.001), Hypothesis 1 was supported. Meanwhile, social exclusion negatively predicted basic psychological needs(*β* = − 0.39, SE=0.02, *p* < 0.001); basic psychological needs positively predicted meaning in life(*β* = 0.53, SE= 0.04, *p* < 0.001). Table 4 further presents the results of the bootstrapping test, confirming that basic psychological needs partially mediated the relationship between social exclusion and meaning in life, with a 95% CI of [-0.21, − 0.08] excluding 0. The direct predictive effect (-0.14) accounted for 40% of the total effect, while the mediated predictive effect (-0.21) accounted for 60% of the total effect, which confirmed Hypothesis 2.

**Table 3:** Mediation analysis results

	M1(criterion: basic psychological needs)				M2 (criterion: meaning in life)			
	β	SE	*p*	95% CI	β	SE	*p*	95% CI
Control Variables								
Gender	0.01	0.02	0.66	[-0.03, 0.04]	− 0.02	0.03	0.51	[-0.07, 0.03]
Grade	0.01	0.01	0.19	[-0.01, 0.03]	− 0.002	0.01	0.90	[-0.03, 0.02]
Independent variable								
Social exclusion	− 0.39^***^	0.02	0.00	[-0.44, − 0.35]	− 0.14^***^	0.03	0.00	[-0.21, − 0.08]
Mediator								
Basic psychological needs					0.53^***^	0.04	0.00	[0.45, 0.61]
* R* ^*2*^	0.24				0.24			
* F*	109.22^***^				83.65^***^			

*p < 0.05; **p < 0.01, ***p < 0.001

**Table 4:** Direct, indirect, and total effects in the mediation model

	Effect	SE	*p*	95% CI
Total effect of Social exclusion on Meaning in life	− 0.35	0.03	0.00	[-0.41, − 0.29]
Direct effect of Social exclusion on Meaning in life	− 0.14	0.03	0.00	[-0.21, − 0.08]
Indirect effect(s) of Social exclusion on Meaning in life	− 0.21	0.02	0.00	[-0.25, − 0.17]

**Fig. 2:**
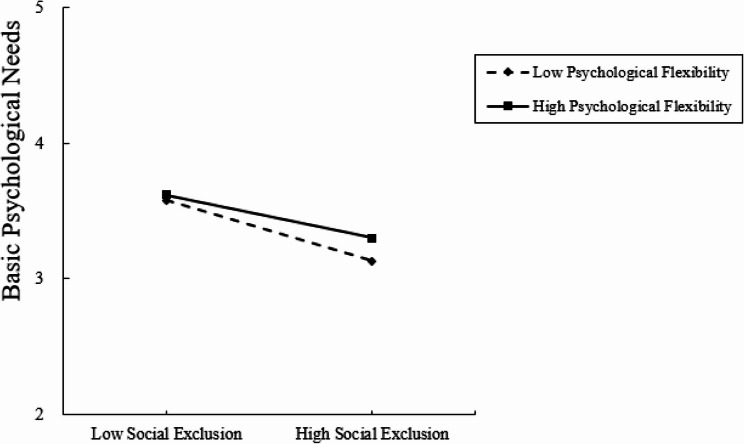
Interaction of social exclusion and psychological flexibility on basic psychological needs

In the second phase, we tested the moderated mediation model with Model 7 of the PROCESS. As presented in Table 5, the interaction between social exclusion and psychological flexibility significantly predicted basic psychological needs (Social exclusion × Psychological flexibility: *β* = 0.11, SE= 0.03, *p* < 0.01) after psychological flexibility was entered into the model. This finding indicates that psychological flexibility moderates the relationship between social exclusion and basic psychological needs. To further examine the moderation pattern of psychological flexibility, we obtained Fig. 2 based on the simple slope test suggested by Aiken and West [41]. The figure displayed the interaction effect at different levels of psychological flexibility (i.e., + 1 SD or -1 SD). Figure 2 indicated this moderating effect: social exclusion was more negatively associated with basic psychological needs when psychological flexibility was low rather than high. Consequently, hypothesis 3 was validated.

**Table 5:** Moderated mediation effect analysis

	M1(criterion: basic psychological needs)				M2 (criterion: meaning in life)			
	β	SE	*p*	95% CI	β	SE	*p*	95% CI
Control Variables								
Gender	0.01	0.02	0.78	[-0.03, 0.05]	− 0.02	0.03	0.51	[-0.07, 0.04]
Grade	0.01	0.01	0.23	[-0.01, 0.03]	− 0.002	0.01	0.90	[-0.03, 0.02]
Independent variable								
Social exclusion	− 0.74^***^	0.11	0.00	[-0.96, − 0.52]	-14^***^	0.03	0.00	[-0.21, − 0.08]
Mediator								
Basic psychological needs					0.53^***^	0.04	0.00	[0.45, 0.61]
Moderator								
Psychological flexibility	− 0.20^*^	0.09	0.03	[-0.38, − 0.02]				
Interaction term								
Social exclusion× Psychological flexibility	0.11^**^	0.03	0.00	[0.04, 0.20]				
* R* ^*2*^	0.26				0.24			
* F*	72.62^**^				83.75^**^			

*p < 0.05; **p < 0.01, ***p < 0.001

Further, Edwards and Lambert’s conditional indirect effects were used to test hypothesis 4 [42]. The conditional indirect effect of social exclusion through basic psychological needs on the meaning in life at different levels of psychological flexibility was assessed by bootstrapping the bias-corrected CIs. As shown in Table 6, the indirect effect of social exclusion on meaning in life via basic psychological needs was stronger and more significant at low levels of psychological flexibility (effect = − 0.24, 95% CI [-0.30, − 0.20]), but weaker at high levels of psychological flexibility (effect = − 0.18, 95% CI [-0.23, − 0.14]). Hence, hypothesis 4 was confirmed.

**Table 6:** Conditional indirect effects across levels of psychological flexibility

Level	Effect	SE	*p*	95% CI
*M-SD*	− 0.24	0.03	0.00	[-0.30, − 0.20]
*M*	− 0.22	0.02	0.00	[-0.26, − 0.17]
*M + SD*	− 0.18	0.02	0.00	[-0.23, − 0.14]

## Discussion

### Results analysis

Grounded in Basic Psychological Needs Theory, the present study investigates the impact of social exclusion on college students’ sense of meaning in life, with a particular focus on the mediating role of basic psychological needs.

First, the results indicate that social exclusion exerts a significant and deleterious influence on college students’ meaning in life. Social exclusion is prevalent among adolescents, who are in a critical stage of developmental vulnerability, both physically and psychologically. Exclusionary experiences can deplete coping resources, engender anxiety, foster social alienation, and undermine existential purpose—ultimately eroding the courage to confront life’s challenges. Furthermore, social exclusion deprives individuals of valuable social resources necessary for maintaining connections with the broader community [43], thereby threatening their perceived life meaning [44, 45]. This finding is consistent with prior research demonstrating that social exclusion precipitates intense feelings of depression, helplessness, and worthlessness [13].

Second, the analysis reveals that basic psychological needs partially mediate the relationship between social exclusion and meaning in life. As a negative interpersonal experience, social exclusion directly impairs the satisfaction of individuals’ basic psychological needs [18], thereby diminishing meaning in life. This outcome resonates with previous evidence that peer interactions serve as an important source of need fulfillment. Zhang et al. further argued that basic psychological needs constitute a fundamental motivational basis for the construction of meaning in life [46]. When these needs are adequately met, individuals are more likely to develop a positive and coherent life meaning; in contrast, the persistent frustration of such needs fosters self-doubt and perceptions of emptiness and meaninglessness—patterns echoed in the current findings.

Finally, the results demonstrate that psychological flexibility moderates the relationship between social exclusion and basic psychological needs. By testing a moderated mediation model, the study shows that psychological flexibility attenuates the mediating effect of basic psychological needs in the pathway between social exclusion and meaning in life. Specifically, individuals with high psychological flexibility can buffer the detrimental impact of exclusion on need satisfaction [47], thereby sustaining a stronger sense of life value and meaning. Conversely, low psychological flexibility amplifies need frustration, magnifying the erosion of meaning in life through unmet psychological needs.

### Theoretical implications

First, while previous research has largely focused on the negative impacts of social exclusion at the emotional and behavioral levels, it has relatively overlooked the critical role of positive psychological resources and adaptive coping strategies. By integrating Basic Psychological Needs Theory with the perspective of psychological flexibility, this study systematically investigates “how” and “under what conditions” social exclusion affects the meaning in life among college students, successfully shifting the research focus from overt behavioral reactions to deep-seated meaning construction. Moreover, the findings lend empirical support to the temporal need–threat model, which posits that socially ostracized individuals suffer from a relative deficit in social connectedness and a reduced sense of purpose [13].

Second, although considerable attention has been devoted to the construct of meaning in life, scholarly investigation into its influencing factors remains limited. Drawing upon Basic Psychological Needs Theory, this study explicates the mechanism through which social exclusion shapes the sense of meaning in life and confirms the central function of basic psychological needs within this process. The findings further emphasize the pivotal role of belonging—as embedded in supportive social relationships—in fostering meaning in life. In the absence of belonging, individuals are prone to experience meaninglessness [17], a conclusion that aligns with King’s [9] tripartite framework for cultivating meaning through belonging, purposeful action, and self-understanding. Accordingly, the study provides a novel theoretical perspective for identifying and analyzing the antecedents that contribute to the development and maintenance of meaning in life.

Third, the empirical results confirm that psychological flexibility exerts a significant moderating effect within the framework of Basic Psychological Needs Theory. This finding not only extends the boundary conditions under which social exclusion manifests its deleterious impact but also reveals the profound value of psychological flexibility as a core resource for sustaining a sense of meaning in life [25]. Individuals with high psychological flexibility are able to maintain an open and flexible cognitive stance, which prevents social exclusion from escalating into a total frustration of basic psychological needs [26]. Consequently, this mitigates the impact on their sense of meaning in life [27]. In conclusion, these findings offer a novel individual differences perspective on the formation mechanisms of meaning in life, elucidating how internal regulatory capacities shape the heterogeneous reconstruction paths of existential well-being amidst social adversity.

### Practical implications

First, the present study offers important empirical insights into the deleterious effects of social exclusion on college students’ sense of meaning in life. Higher levels of perceived exclusion are associated with an increased likelihood of losing confidence, purpose, and perceived self-worth. These findings underscore the need for educators and university administrators to foster inclusive and supportive campus environments, proactively identify and engage students experiencing exclusion, and ensure accessible channels for help-seeking. Moreover, it is recommended that educators provide timely psychological counseling, constructive guidance, and preventive interventions for vulnerable students. Such measures can mitigate the adverse psychological consequences of social exclusion, thereby reducing the incidence of meaninglessness among college students.

Second, empirical results indicate that the indirect effect of basic psychological needs is − 0.21, accounting for as high as 60% of the total effect. This suggests that any external intervention aimed at restoring meaning must first repair the individual’s sense of autonomy, competence, and relatedness, as these are the non-negotiable precursors to a purposeful life. Educators must broaden their focus beyond students’ academic performance, recognize the multifaceted nature of students’ needs, and provide ample resources and opportunities to cater to them. Teachers should collaborate to offer the necessary interpersonal support to ensure that students’ basic psychological needs are met, resulting in higher levels of meaning in their lives.

Finally, the empirical results indicate that under conditions of low psychological flexibility, the negative indirect effect of social exclusion on meaning in life through basic psychological needs is significantly more pronounced. This finding suggests that we should cultivate students’ psychological flexibility in a targeted manner and provide additional support to those with lower levels of flexibility. Educators should transition from general psychological support to precision-guided interventions, such as Acceptance and Commitment Therapy (ACT). By training students in psychological flexibility, universities can help vulnerable individuals “unhook” from the immediate pain of exclusion, thereby safeguarding their core psychological needs and maintaining their sense of meaning in life.

### Limitations and future research

First, this study primarily relies on self-report questionnaires, which substantially constrains the strength of causal inference among variables. Although we adopted a multi-wave design, the inherent limitations of questionnaire-based methods, such as common method bias and social desirability effects, still persist. Future research that integrates experimental designs or employs objective measures would contribute to further validating the causal relationships proposed in this study and exploring other potential causal pathways. Second, due to constraints of time, workforce, and resources, the scope of this study was limited. To enhance the external validity of the findings, future research can seek to expand the sample’s source and scope. This could include investigating the phenomenon in different cultural contexts or with larger and more diverse samples. Third, this study found that basic psychological needs partially mediate the relationship between social exclusion and meaning in life, as well as the moderating role of psychological flexibility. This suggests the potential existence of other mediating variables and boundary conditions. For instance, future studies could examine the mediating effects of factors such as emotion regulation, depressive mood, and social identity; alternatively, they could explore how boundary conditions like self-compassion, mindfulness, and teacher support influence this mechanism.

## Data Availability

Data will be available on request.

## Abbreviations

ACT: Acceptance and Commitment Therapy
AAQ-II: Acceptance and Action Questionnaire-Second Edition
BPN: Basic Psychological Needs
BPNS: Basic Psychological Needs Scales
MLQ: Meaning in Life Questionnaire
OES-A: Ostracism Experience Scale for Adolescents
M: Mean
SD: Standard Deviation
CFI: Comparative Fit Index
TLI: Tucker-Lewis Index
RMSEA: Root Mean Square Error of Approximation
CI: Confidence Interval
SE: Social Exclusion
